# A *Dunaliella salina* Extract Counteracts Skin Aging under Intense Solar Irradiation Thanks to Its Antiglycation and Anti-Inflammatory Properties

**DOI:** 10.3390/md20020104

**Published:** 2022-01-27

**Authors:** Fabien Havas, Shlomo Krispin, Moshe Cohen, Estelle Loing, Morgane Farge, Thierry Suere, Joan Attia-Vigneau

**Affiliations:** 1 International Flavors & Fragrances—Lucas Meyer Cosmetics, Faran 4, Yavne 8122503, Israel; fabien.havas@iff.com (F.H.); shlomo.krispin@iff.com (S.K.); moshe.cohen@iff.com (M.C.); 2 International Flavors & Fragrances, 521 West 57th Street, New York, NY 10019, USA; estelle.loing@iff.com; 3 International Flavors & Fragrances—Lucas Meyer Cosmetics, 195 Route d’Espagne, 31 036 Toulouse, France; morgane.farge@lucasmeyercosmetics.com (M.F.); thierry.suere@lucasmeyercosmetics.com (T.S.)

**Keywords:** algae, skin aging, glycation, inflammation, antiaging, sun care, carotenoid, phytoene, phytofluene, *Dunaliella*

## Abstract

Glycation, and the resulting buildup of advanced glycation end products (AGEs), is recognized as a key driver of cumulative skin damage and skin aging. *Dunaliella salina* is a halophile microalga adapted to intense solar radiation through the production of carotenoids. We present a natural supercritical CO_2_ extract of *Dunaliella salina* rich in the colorless carotenoids phytoene and phytofluene. The extract exhibited antiglycation and anti-inflammatory activities in ex vivo testing, showing strongly reduced formation of N-ε-carboxy-methyl-lysine with exposure to methylglyoxal, reduced AGE receptor levels, and significantly reduced interleukins 6 and 8. In a placebo-controlled clinical study under intense solar exposure, the extract significantly reduced the skin’s glycation scores and its sensitivity to histamine; key skin aging parameters were also significantly improved vs. placebo, including wrinkle counts and spots. These results demonstrate the value of this *Dunaliella salina* extract, rich in colorless carotenoids, as an antiglycative, anti-inflammatory, and antiaging active ingredient, including in high-irradiation contexts.

## 1. Introduction

Aging may be defined as the progressive accumulation of damage to living tissue over the organism’s lifetime. Skin is subjected both to internal aging processes and to various external stressors, leading to distinct structural changes affecting its youthful appearance and its vital physiological functions. Aged skin is characterized by loss of elasticity, wrinkles, dryness, reduced thickness/atrophy, reduced cell proliferation, and cellular senescence [1]. External factors contributing to skin aging include sunlight, UV radiation, chemicals, pollutants, and smoking.

In recent years, a large body of work has shown that advanced glycation end products (AGEs) are also among the crucial contributing factors of skin aging [2]. The formation of AGEs is a very complex process involving a spontaneous nonenzymatic reaction known as glycation. Glycation is a reaction between reducing sugars on the one hand and proteins, lipids, or nucleic acids on the other. In glycation, reactive carbonyl groups belonging to a reducing sugar react with free amino groups on proteins to form an unstable Schiff base. Rearrangement of the Schiff base then results in the formation of more stable ketoamines known as Amadori products. These reversible reaction products subsequently undergo irreversible oxidation, polymerization, dehydration, and cross-linking reactions to generate AGEs [3]. The progressive accumulation of these AGEs in the body is now recognized as a hallmark of the aging process. The most commonly found AGEs in the skin include carboxymethyl-lysine (CML), carboxyethyl-lysine, and fructose-lysine. Receptors for AGEs (RAGEs) are generally expressed in the epidermis and dermis, and it has been observed that their expression is higher in the sun-exposed areas of the skin as compared with the sun-protected areas [4].

Accumulation of AGEs in the skin has been observed both in diabetes and during chronological aging. Proteins with a slow turnover rate, such as collagens I and IV, as well as long-lived proteins, such as fibronectin, are primary targets of glycation reaction in the skin. Moreover, excessive deposition of AGEs in sun-exposed skin areas suggests that solar radiation, especially UV radiation, may play an important role in the formation of AGEs. AGEs generated by smoking or ingested from diet may also participate in skin aging [5,6,7].

Glycation of biological molecules modifies their biomechanical and functional properties. Proteins, lipids, and nucleic acids can be targets of glycation, modifying protein conformation and solubility, enzyme–substrate interactions, protein–DNA interactions, protein–protein interactions, DNA regulation, and epigenetic modulation, thus interfering with numerous physiological functions of the targets of glycation [8]. For example, glycation of collagen or elastin and resulting cross-linking can lead to increased stiffness and resistance to removal by matrix metalloproteinases (MMPs) [9]. Moreover, RAGE activation has been shown to result in inflammatory and immune responses, increased MMP production, impaired cell proliferation, excessive melanogenesis, and altered gene expression [10,11,12,13]. All of these processes can potentially contribute to skin aging.

Biological mechanisms contributing to the detoxification of AGEs include the vital glyoxalase enzymatic pathway, including the enzyme glyoxalase-1 (Glo-1), whose main substrate is methylglyoxal (MG), a widely recognized initiator of glycation formed in animal tissues as a byproduct of glycolysis and other metabolic processes [14,15,16,17]. However, in light of the literature record presented above, it is evident that these mechanisms are, unaided, unequal to the task of controlling glycation and its effects.

In light of the involvement of AGEs in aging and age-related disorders, potential strategies for prevention or treatment have been put forward, including inhibiting the formation of AGEs, removing existing AGEs by degrading glycated proteins, and antagonizing the AGE-mediated signaling cascade. Some synthetic AGE inhibitors have been put forward, but so far with relatively low efficacies, poor pharmacokinetics, or unsatisfactory safety profiles. Natural compounds including phenolics, oligo- and polysaccharides, carotenoids (e.g., β-carotene), and unsaturated fatty acids have been reported to possess antiglycating activity [18]. Among these, the ability of some microalgal extracts to inhibit AGE formation differs from that of many other botanicals, in that it is not being promoted by phenolic compounds. A wide range of antioxidant compounds can be found in microalgae, including carotenoids and polyunsaturated fatty acids, such as linoleic acid, arachidonic acid, and eicosapentaenoic acid [19].

*Dunaliella salina* is a halophile unicellular microalga found in high-salinity environments (salt beds and lakes, evaporation ponds; though the alga is capable of surviving in lower-salinity environments, it is especially well adapted to high salinities and appears to be rarer in the general marine environment) [20]. Under stress (from high solar irradiation), this alga adapts by generating large amounts of β-carotene and its precursors, the colorless carotenoids phytoene and phytofluene [21]. The worldwide commercial cultivation of *Dunaliella* to produce β-carotene is now one of the success stories of halophile biotechnology. Different technologies are used, from low-tech extensive cultivation in lagoons to intensive cultivation at high cell densities under controlled conditions. One of the methods used in such biotechnological operations to induce massive carotenoid accumulation is the reduction of the growth rate by nutrient deprivation in combination with high light intensities [22]. *Dunaliella salina* also contains ω-3 unsaturated eicosapentaenoic acid (EPA), which is not known in any terrestrial carotenogenic plant. EPA, along with other unsaturated fatty acids, such as linoleic and linolenic acids, has known antiglycation properties [23].

**Figure 1:**
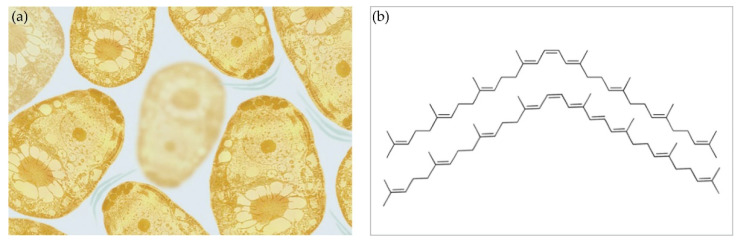
(**a**): *Dunaliella salina*, illustrative microscopy image. (**b**): phytoene (top) and phytofluene (bottom).

Some carotenoids, especially lutein, β-carotene, and astaxanthin, have been shown to have antiglycative power [24]. However, the use of most carotenoids in topical cosmetic formulations remains difficult due to the usually intense color imparted to the formula and to the skin upon application. This is not the case, on the other hand, with the colorless carotenoids phytoene and phytofluene. Phytoene and phytofluene are the biosynthetic precursors for all colored carotenoids and, due to their shorter conjugated C=C double-bond chromophores, absorb light in the UV range and not in the visible range. The colorless carotenoids are found in most carotenogenic organisms, including the unicellular alga *Dunaliella salina* (Figure 1). These molecules have beneficial properties useful in health, nutritional, and cosmetic applications. This includes a measure of protection against UV and oxidative damage leading to premature aging and other disorders. The colorless carotenoids have been shown to have antioxidative, anti-inflammatory, and DNA protection activities [25]. While no evidence has been published to date on this effect, the colorless carotenoids may thus hold some promise for protection from glycation-induced aging and solar damage.

We therefore present here results showing that a hydrophobic extract of *Dunaliella salina*, standardized in phytoene and phytofluene, is an effective botanical active ingredient for the protection of skin against some effects of solar exposure, helping to mitigate glycation and its associated effects on skin, including inflammation, and the appearance of signs of aging (wrinkles).

## 2. Results

### 2.1. Dunaliella Salina Extract

**Figure 2:**
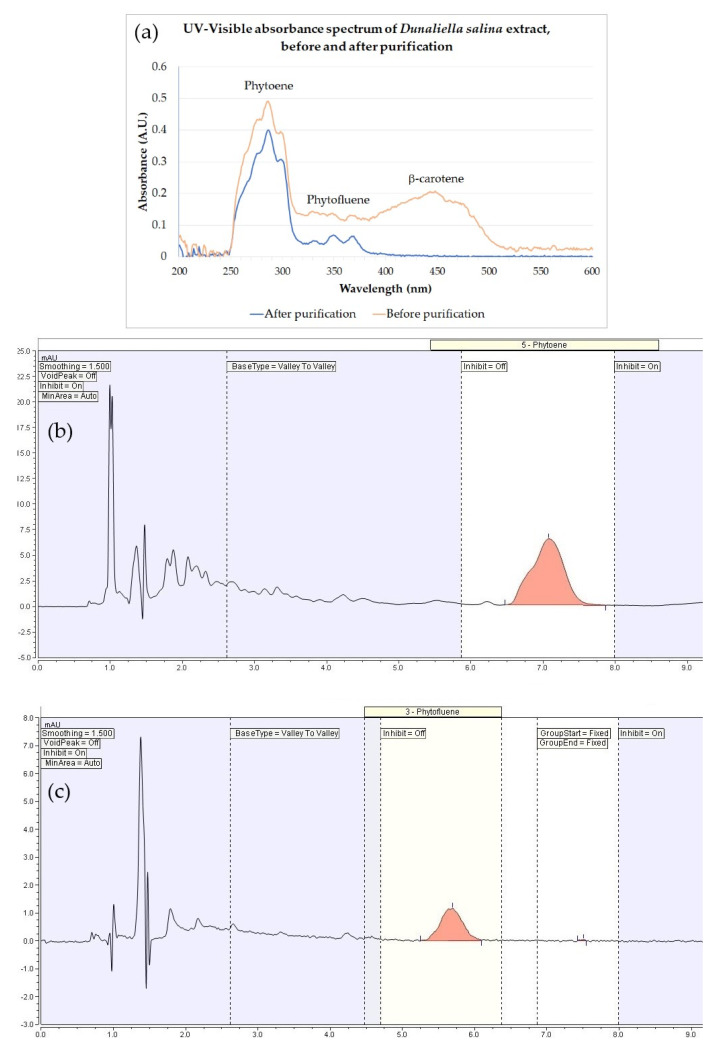
Analytical characterization of the extract and detection of phytoene and phytofluene. (**a**): UV–visible spectroscopy showing the peaks for phytoene (286 nm), phytofluene (348 nm), and β-carotene (447 nm) in the extract before purification, and the absence of β-carotene in the extract after purification, while retaining peaks for phytoene and phytofluene. (**b**): HPLC–DAD trace, phytoene. (**c**): HPLC–DAD trace, phytofluene.

The studies described herein employed a hydrophobic extract of *Dunaliella salina* (IBR-Solage^®^, International Flavors & Fragrances—Lucas Meyer Cosmetics, Yavne, Israel) obtained by supercritical CO_2_ extraction followed by purification to remove colored components including β-carotene, while preserving the colorless carotenoids phytoene and phytofluene in the extract. The extract was standardized to 0.025 mg/mL phytoene and phytofluene by dilution in jojoba seed oil. While before the purification step the extract possessed a strong yellow-orange coloration, the purified extract’s final appearance was that of an essentially colorless to palest yellow oil. This is reflected in the colorimeter readings shown in Table 1, showing a dramatic decrease in the b* value (loss of the yellow coloration), accompanied by a rise in L* (lightening) and a drop in a* (reduced redness). The same can be observed from the UV–visible spectra (Figure 2), showing the removal of the β-carotene peak (447 nm) as well as that of other minor components absorbing light in the visible range, while preserving the phytoene and phytofluene peaks (286 and 348 nm, respectively). Detection of phytoene and phytofluene by HPLC–DAD is also shown in Figure 2.

### 2.2. Antiglycation Effect in Skin Explants

The antiglycation effect of the *Dunaliella salina* extract was tested in a human skin ex vivo model under basal conditions and following stimulation of glycation with the widely recognized glycation initiator, methylglyoxal.

**Figure 3:**
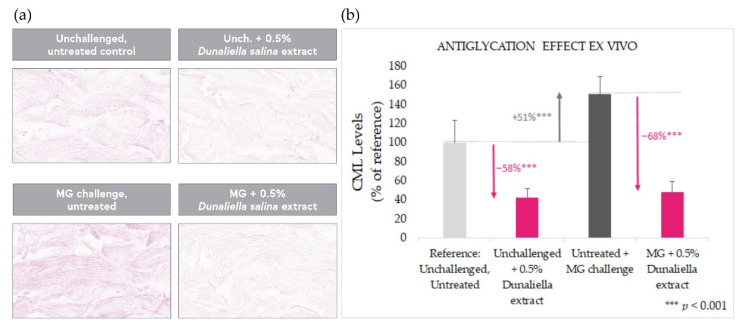
Antiglycation effect in skin explants with and without challenge by MG, evaluated by CML staining. (**a**): illustrative images. (**b**): quantitative image analysis results normalized to the unchallenged, untreated baseline, showing reduced CML levels upon treatment with the extract.

As expected, methylglyoxal stimulation gave rise to a significant increase (+51%, *p* < 0.001) in glycation, as evaluated by immunostaining of N-epsilon-(carboxymethyl)lysine (CML), a common AGE and a classic glycation marker (Figure 3). Treatment with *Dunaliella salina* extract over 10 days results in a strong antiglycation effect, both in unchallenged explants (*p* < 0.001) and in explants challenged with methylglyoxal (*p* < 0.001)—most pronounced in the latter (up to −68%).

**Figure 4:**
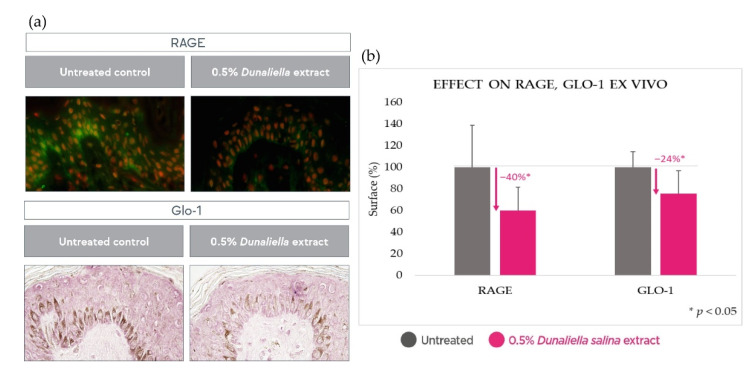
Reduction of RAGE and Glo-1 in skin explants upon treatment with the *Dunaliella* extract compared with untreated baseline. (**a**): illustrative images. (**b**): quantitative image analysis results.

Additionally, in human skin explants, 10 days’ treatment with the *Dunaliella salina* extract caused a significant reduction (*p* < 0.05) in both RAGE and glyoxalase-1 by 40% and 24%, respectively, further corroborating a reduced glycation state following treatment with the extract (Figure 4).

### 2.3. Anti-Inflammatory Effect in Skin Explants

**Figure 5:**
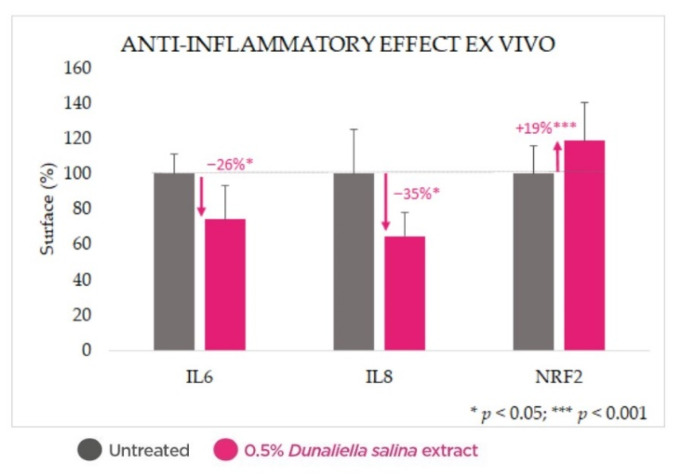
Anti-inflammatory effect ex vivo: reduction of interleukins 6 and 8 and increased NRF2 upon treatment with the *Dunaliella* extract compared with untreated baselines.

Treatment of skin explants with 0.5% *Dunaliella salina* extract for 24 h resulted in significant reductions in levels of inflammatory interleukins 6 and 8 (by 26% and 45%, respectively, *p* < 0.05), as well as significant enhancement of the key antioxidant and anti-inflammatory regulator nuclear factor erythroid 2-related factor 2 (NRF2) (by 19%, *p* < 0.001) (Figure 5).

### 2.4. Clinical Evaluation of Protective Effects under Intensive UV Irradiation

The extract was incorporated into a simple cream gel chassis, which was tested over 8 weeks in a double-blind, split-face, placebo-controlled clinical trial on 25 female volunteers aged 35–60. Panelists were expected to undergo daily intense solar exposure over the course of the study (defined as beachgoing or similar activities), which was carried out in Portugal during the peak summer months.

#### 2.4.1. Antiglycation Effect

**Figure 6:**
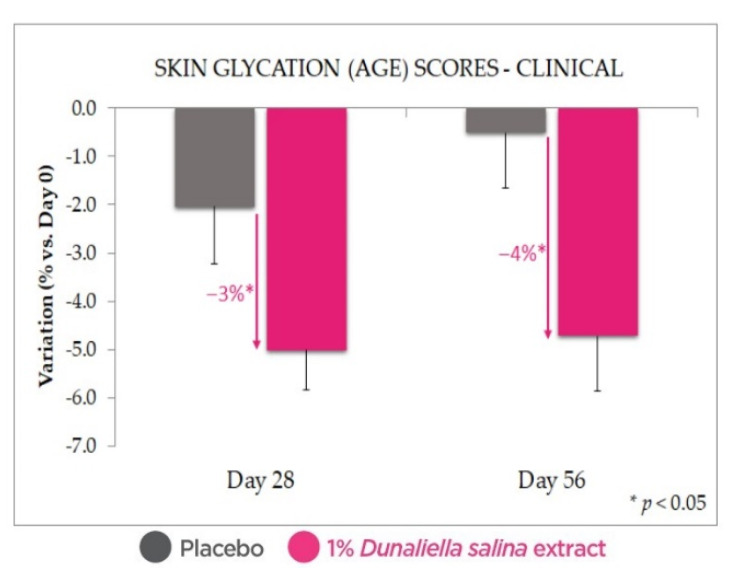
Antiglycation effect in vivo: reduced skin glycation scores with 1% *Dunaliella* extract in formulation compared with placebo. Standard deviation bars were scaled by a factor of 10 for readability.

Glycation score measurements (AGE reader, Figure 6) confirm the antiglycative indication observed in the ex vivo data shown above, with a statistically significant reduction in glycation scores with 1% *Dunaliella salina* extract in formulation, at both Days 28 and 56 (and with *p* < 0.05 vs. Day 0 and vs. placebo). While the AGE scores measured with the placebo at Day 56, and especially at Day 28, were lower than those at Day 0, it should be noted that the difference vs. Day 0 was not statistically significant.

#### 2.4.2. Anti-Inflammatory Effect

**Figure 7:**
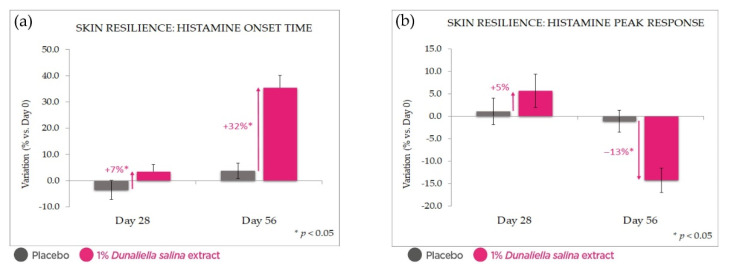
Skin resilience improvement in vivo: microcirculation as a measure of the inflammatory response to histamine stimulation. (**a**): onset time of reaction to histamine. (**b**): peak response intensity. Both show an attenuated reaction with 1% *Dunaliella* extract in formulation, compared with placebo and Day 0. Standard deviation bars were scaled by a factor of 10 for readability.

In order to assess the skin’s resilience to inflammatory insult, the reaction to stimulation by histamine (a chemical insult commonly used as a model for this purpose) was evaluated via measurement of skin microcirculation. After 56 days of treatment, we observed a strong reduction of the skin’s reactivity to histamine stimulation (Figure 7). This was expressed in an increase of over 35% in reaction onset time and a 14% reduction in the amplitude of induced microcirculation vs. Day 0; compared with placebo, treatment with the extract led to a 32% higher reaction time and 13% lower amplitude of reaction (all with *p* < 0.05).

**Figure 8:**
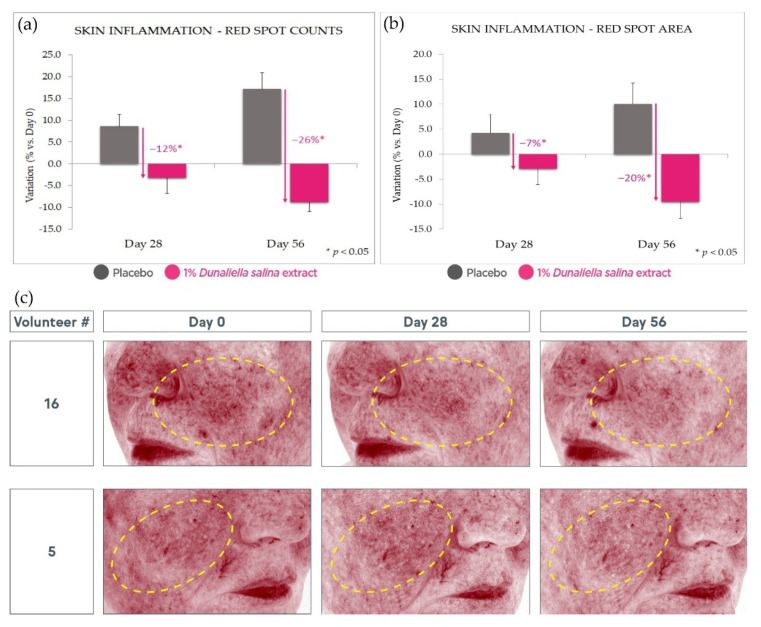
Red spot reduction. (**a**): red spot count reduction with 1% extract in formulation compared with placebo and Day 0. (**b**): similarly, red spot area reduction. (**c**): illustrative photographs. Standard deviation bars were scaled by a factor of 10 for readability.

At the same time, 1% *Dunaliella salina* extract in formula showed a strong advantage over placebo in an evaluation of red spot counts and areas by Visia image analysis (at Day 56, −26% and −20%, respectively, vs. placebo, *p* < 0.05) (Figure 8). While the placebo exhibits a significant increase in red spots expected under intense solar exposure, the active product negates and even reverses this effect, leading to a decrease vs. Day 0. Some of these effects can already be observed at Day 28, albeit with a lower magnitude and statistical significance.

#### 2.4.3. Antiaging Effect

**Figure 9:**
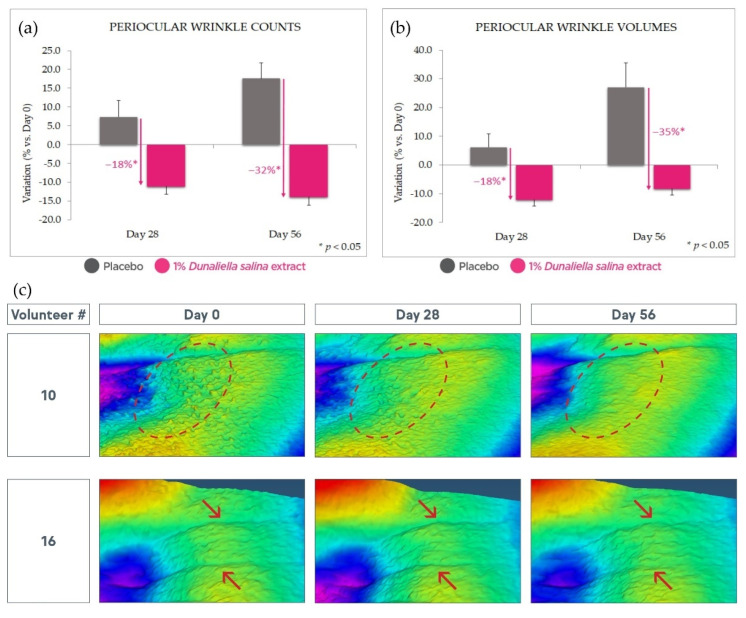
Antiaging effect. (**a**): wrinkle count reduction with 1% extract in formulation compared with placebo and Day 0. (**b**): similar wrinkle volume reduction. (**c**): illustrative images; circles and arrows emphasize features of interest (skin roughness and wrinkles, respectively). Standard deviation bars were scaled by a factor of 10 for readability.

Finally, after 56 days’ use, 1% *Dunaliella salina* extract displayed a strong antiaging effect, as manifested in a significant reduction of wrinkle counts and volume (respectively, ca. 32% and 35% vs. placebo, *p* < 0.05) as evaluated by Aeva image analysis (Figure 9). These effects are also observable at Day 28 with lower magnitude and statistical significance.

## 3. Discussion and Conclusions

The results of the ex vivo studies presented above indicate that the colorless carotenoid-containing *Dunaliella* extract introduced here indeed possesses the antiglycation properties hypothesized based on the literature reviewed. In our studies, these were expressed in reductions of RAGE and Glo-1 upon treatment with the extract, and in strongly reduced levels of the glycation marker CML, with and without stimulation of glycation by methylglyoxal.

An additional explant model confirmed the expected anti-inflammatory properties of the colorless carotenoid-containing *Dunaliella* extract, expressed as reductions in key interleukins, IL6 and IL8, and increased NRF2.

These effects were confirmed in a clinical trial under intense solar exposure, where 1% *Dunaliella salina* extract in formulation reduced glycation scores in comparison with placebo, strengthened the skin’s resilience to irritation and its capacity to fight inflammation-inducing provocations (such as the histamine model insult used here), and strongly reduced skin redness and wrinkles compared with Day 0 and even more so when compared with placebo.

It is worth noting that a worsening in red spot and wrinkling parameters was observed with the placebo product. This might be predicted based on the study’s high solar exposure conditions, which are expected to induce photodamage in exposed skin. In that context, we observed that the active product not only negated this effect (protecting the skin), but reversed it, producing significant improvements vs. the initial state despite hostile conditions.

The understanding of these effects could be expanded in future studies by examining the dose dependency of the effects demonstrated here, in the same or similar ex vivo and clinical models. In addition, examination of the mechanisms involved might profitably be broadened by testing these effects at different time points, in particular regarding the effects on RAGE and Glo-1 in skin explants. Finally, the effect of the extract on RAGE and Glo-1, and arguably on inflammation markers as well, might be re-examined under stimulation by methylglyoxal, in addition to the basal state presented above.

Nevertheless, we believe that the results presented here effectively demonstrate the value of this *Dunaliella salina* extract as an active ingredient in cosmetic applications aimed at protecting the skin from damage and premature aging, including under intense solar exposure conditions.

## 4. Materials and Methods

### 4.1. Extract

The studies described herein employed a hydrophobic extract of *Dunaliella salina* (IBR-Solage^®^, International Flavors & Fragrances – Lucas Meyer Cosmetics, Yavne, Israel). The alga was cultivated in shallow open raceway ponds in Spain. After an initial population growth stage, production of carotenoids was promoted by means including nutrient deprivation (in addition to naturally high solar radiation). After harvesting by centrifugation, the alga was freeze-dried and extracted with supercritical CO_2_, and the resulting oleoresin was taken up in a carrier oil (colorless *Simmondsia chinensis* (jojoba) seed oil [26], Jojoba Israel, Kibbutz Hatzerim, Israel 110200180) and further purified by treatment with an adsorbent (montmorillonite bleaching earth) to remove colored components, retaining the colorless carotenoids. The final extract was standardized for content in the key colorless carotenoids phytoene and phytofluene (0.025 mg/mL) by dilution in jojoba oil based on quantification by HPLC–DAD and UV–visible spectroscopy, yielding an essentially colorless oil [27,28,29]. Carotenoid standards were purchased from the Lycored Company (Beer Sheva, Israel).

UV–visible spectrometry: The extract was diluted ×5 in hexanes (95% n-hexane, HPLC grade, J.T. Baker 9304-02, Avantor, Radnor, PA, USA) and measured against a similarly diluted jojoba oil blank sample (Jojoba Israel, as above); the spectrum was recorded in absorbance mode from 200 to 600 nm (Spectroquant Pharo 300, Merck, Burlington, MA, USA). The phytoene absorbance peak was measured at 286 nm, and the phytofluene peak at 348 nm.

HPLC–DAD chromatography employed a Dionex UltiMate 3000 with DAD 3000 diode array detector (Thermo Fisher Scientific, Waltham, MA, USA) and Kinetex 00D-4462-E0 2.6 µm C18 100 Å 100 × 4.6 mm column (Phenomenex, Torrance, CA, USA). The extract was diluted ×5 in hexanes (J.T. Baker, as above) and filtered (0.22 μm) before injection. Injection volume: 10 μL; flow: 1.0 mL/min; column temperature: 40 °C; detection wavelengths: 286 nm (phytoene), 348 nm (phytofluene); eluent: acetonitrile (J.T. Baker 9017, Avantor, Radnor, PA, USA)/dichloromethane (J.T. Baker 9315-02, Avantor, Radnor, PA, USA)/hexanes (J.T. Baker, as above)/methanol (J.T. Baker 8402, Avantor, Radnor, PA, USA), 19/1/1/19 by volume +0.05% diisopropylethyl amine (Sigma-Aldrich 8.00894, St. Louis, MO, USA).

Extract color was measured as an L,a,b tricolor stimulus using a ColorFlex EZ colorimeter (HunterLab, Reston, VA, USA) standardized to 0.025 mg/mL phytoene and phytofluene but without further sample dilution (neat final extract or its equivalent at earlier processing stages).

### 4.2. Antiglycation Effect of the Extract on Human Skin Explants

This study was performed on human skin tissue obtained from surgical residues in full respect of the Declaration of Helsinki and Article L.1243-4 of the French Public Health Code. The latter does not require any prior authorization by an ethics committee for sampling and using surgical wastes.

Normal human skin explants (12 mm) from a 34-year-old woman were incubated in survival culture medium at 37 °C, 5% CO_2_ in a humidified atmosphere. A group of explants were left untreated, while another was treated topically with 2 mg/cm^2^ (2 μL/explant) of a 0.5% solution of the extract in jojoba oil (jojoba Israel, as above) on Days 3, 4, 5, and 7. In order to induce glycation, half of each group was challenged with 500 μM methylglyoxal (Sigma-Aldrich M0252, St. Louis, MO, USA) via the culture medium from Day 4, while the other half remained unchallenged. Fifty percent of the culture medium was replaced with fresh medium on Days 3, 5, and 7.

On Day 0, 3 reference explants were collected and cut in 2 parts. Half was fixed in buffered formalin solution, and half was frozen at −80 °C. On Day 10, 3 explants of each group were collected and processed in the same way. After fixation for 24 h in buffered formalin, the samples were dehydrated and impregnated in paraffin using a Leica PEARL tissue processor (Leica Biosystems, Buffalo Grove, IL, USA). The samples were embedded using a Leica EG 1160 embedding station (Leica Biosystems, Buffalo Grove, IL, USA). Sections that were 5 μm thick were made using a Leica RM 2125 Minot-type microtome (Leica Biosystems, Buffalo Grove, IL, USA), and the sections were mounted on Superfrost^®^ histological glass slides (Thermo Fisher Scientific, Waltham, MA, USA). Frozen samples were cut at 7 μm thickness with a Leica CM 3050 cryostat (Leica Biosystems, Buffalo Grove, IL, USA). The sections were then mounted on Superfrost^®^ Plus silanized glass slides (Thermo Scientific, Waltham, MA, USA). The microscopical observations were made using a Leica DMLB (Thermo Scientific, Waltham, MA, USA), or an Olympus BX43 or BX63 microscope (Olympus Life Science, Tokyo, Japan). Pictures were digitized with a numeric DP72 or DP74 Olympus camera with cellSens storing software (Olympus Life Science, Tokyo, Japan).

The cell viability of the epidermal and dermal structures was assessed by microscopic observation of formol-fixed paraffin-embedded skin sections after Masson’s trichrome staining (Goldner variant).

Immunostaining for N-epsilon-(carboxymethyl)lysine (CML), a common AGE and a classic glycation marker, was carried out on fixed skin sections with a monoclonal anti-CML antibody (TransGenic ref. KH011, clone CMS-10, TransGenic, Fukuoka, Japan) diluted at 1:25 in PBS–BSA 0.3% overnight at room temperature using a Vectastain Kit avidin/biotin amplifier system revealed by VIP (Vector Laboratories SK-4600, Burlingame, CA, USA), a substrate of peroxidase generating a violet stain when oxidized.

Immunostaining for RAGE was carried out on frozen skin sections with a polyclonal anti-RAGE antibody (R&D Systems ref. AF1145, Bio-Techne, Minneapolis, MN, USA) diluted at 1:50 in PBS–BSA 0.3% Tween 20 at 0.05% overnight at room temperature. The staining was revealed by Alexa Fluor 488 (Life Technologies ref. A11078, Thermo Fisher Scientific, Waltham, MA, USA). The nuclei were poststained using propidium iodide.

Immunostaining for Glo-1 was carried out on formalin-fixed paraffin-embedded (FFPE) skin sections with a monoclonal anti-Glo-1 antibody (Novus Biologicals ref. H00002739-M01, clone 4C12, Bio-Techne, Minneapolis, MN, USA) diluted at 1:50 in PBS–BSA 0.3% Tween 20 at 0.05% for 1 h at room temperature using a Vectastain Kit Vector avidin/biotin amplifier system revealed by VIP (Vector Laboratories, as above).

Image analysis was used to quantify the staining intensity using the cellSens software (Olympus Life Science, Tokyo, Japan). Pixels corresponding to the staining were selected by thresholding, regions of interest (ROI) were defined by drawing, a combination of these two masks resulted in the selection of the immunostaining within the ROI, and the surface of this immunostained region was expressed as % of the overall ROI.

### 4.3. Anti-Inflammatory Effect of the Extract on Human Skin Explants

This study was performed on human skin tissue obtained from surgical residues (Alphenyx, Marseille, France), in full respect of the Declaration of Helsinki and Article L.1243-4 of the French Public Health Code. The latter does not require any prior authorization by an ethics committee for sampling and using surgical wastes.

Normal skin biopsies (residues from abdominal surgery from three Caucasian female donors aged 28, 38, and 40 years) were cultured according to the method originally described by Trowell and Companjen [30]. Biopsy samples were inserted in a Transwell filter (pore size of 0.4 μm; Greiner Bio-One #665640, Kremsmünster, Austria), punched (2 mm), and placed in a culture plate. The epidermis was held facing upwards at the liquid–air interface, while the dermis was immersed into the culture medium. The explants were then incubated at 37 °C in the presence of 5% CO_2_ in a humidified atmosphere in complete Dulbecco’s Modified Eagle Medium (DMEM) with 1% nonessential amino acids (AA) (Gibco #11140-035, Thermo Fisher Scientific, Waltham, MA, USA). After 24 h, the culture medium was replaced with serum-free DMEM containing 1% antibiotics and 1% AA (Dutscher #L0060-500, Bernolsheim, France; Sigma #P0781, St. Louis, MO, USA) for 8 h. A 0.5% solution of the *Dunaliella* extract in jojoba oil (Jojoba Israel, as above) or jojoba oil only (control) was then added topically for an additional 24 h.

At the end of the incubation period, supernatants were collected, and IL6 and IL8 released from the explants were quantified by enzyme-linked immunosorbent assay (ELISA) (R&D System DY206 and DY208, Bio-Techne, Minneapolis, MN, USA). Results were expressed as percentage of IL6 synthesis normalized to cellular viability.

Tissue samples were frozen at −80 °C, embedded in an optimal cutting temperature (OCT) embedding matrix (Cell Path #KMA-0100-00A, Powys, UK), cut in 5 µm sections, and stored at −20 °C until staining. NRF2 was labeled with a specific antibody (Abcam #ab76026, Cambridge, UK) diluted at 1/200 in PBS–1% BSA and revealed with a secondary antibody (Alexa Fluor 488, Thermo Fisher, Waltham, MA, USA). Microscopic observations were performed using a Zeiss X40 microscope (Carl Zeiss AG, Oberkochen, Germany). Pictures were digitized with a numeric Zeiss camera with Zen lite software (Carl Zeiss AG, Oberkochen, Germany). Four sections were cut for each experimental group in the study, and 5 pictures were taken of each section. Image processing was performed using Fiji software [31]. A DAPI dye (Santa Cruz #sc-24941, Dallas, TX, USA) was used in order to identify nuclei in the epidermis region (blue channel). The green channel was used to quantify GFP fluorescence labeling in the same region. A 2D Gaussian blur filter was used to remove noise. K-means clustering was used to isolate the epidermis ROI and epidermis spots from the background and dermis by partitioning pixels based on intensity. The measurement tool was used to calculate area statistics in the epidermis region and spots. The results are reported as percentage of the total fluorescence intensity, comparing the control and the treated samples.

Data were submitted to the 2-way paired Student *t*-test (* *p* < 0.05, ** *p* < 0.01, *** *p* < 0.001).

### 4.4. Clinical Evaluation of Protective and Antiaging Effects under Intense Solar Exposure

The *Dunaliella salina* extract described above was formulated at 1% into a simple gel cream, along with a corresponding placebo (Table 2), in which the extract was replaced with jojoba oil (Jojoba Israel, as above). These 2 formulations were tested in a double-blind, placebo-controlled trial. All subjects gave their informed consent for inclusion before participating in the study. The study was conducted in accordance with the Declaration of Helsinki, and the protocol was approved by the Ethics Committee of Clinica Dr. Carlos Ramos of Portugal (protocol code PT.06.01, version 3.0, dated 18 October 2019; approval date: 25 October 2019). The 25 female volunteers included in this study were aged 35–60, possessed Fitzpatrick skin phototypes II–IV, and exhibited signs of aging (defined as noticeable wrinkles and fine lines). Panelists were further selected on the basis of expected daily intense solar exposure over the course of the study (defined as beachgoing or similar activities), which was carried out in Portugal during the peak summer months. Products were applied for 56 days following a randomized split-face scheme, where one side of the face received the active product, and the other side received the placebo. Product formulations are summarized in Table 1.

The skin glycation status was assessed using an AGE Reader (DiagnOptics Technologies B.V., Groningen, Netherlands) based on skin autofluorescence. The anti-inflammatory effect was evaluated via laser Doppler flowmetry measurement of skin microcirculation (PeriFlux LDPM PF5000 with a PF 5010 laser channel, Perimed, Jakobsberg, Sweden). Histamine, known to increase capillary permeability and vasodilation and therefore recognized as a reliable model to assess cutaneous susceptibility to inflammation, was dispensed by iontophoresis (Perilont, Perimed, Jakobsberg, Sweden; 30 s at 200 μA). Onset time was calculated as the time between the start of the histamine application and the start of blood flow increase. Maximal blood flow increase was also recorded. The effect on red spots was evaluated by cross-polarized image analysis (Visia-CA, Canfield, Parsippany, NJ, USA). The antiwrinkle effect was evaluated by 3D image analysis (Aeva-HE, Eotech SAS, Marcoussis, France). Measurements were carried out at Days 0, 28, and 56 of the study. All evaluations were performed in 1 of 2 defined time periods of the day (09:00–13:00 or 13:00–19:00, selected for each panelist at the start of the study and kept constant for all subsequent measurements) in a climate-controlled room (temperature = 21 °C ± 2 °C; relative humidity = 55% ± 10%) after an acclimatization process of at least 15 min.

Resulting data and percentage variations were submitted to the paired Student’s *t*-test or the Wilcoxon test (* *p* < 0.05).

## Figures and Tables

**Table 1:** Extract colorimetry data.

Extract	L*	a*	b*
Before purification	60.27	0.07	74.00
After purification	65.49	−1.48	5.59

**Table 2:** Clinical trial product formulations.

Ingredient	% in Formula—Active	% in Formula—Placebo
Water	85.85	85.85
Butylene glycol	4.00	4.00
Dipropylene glycol	1.00	1.00
Hexylene glycol	1.00	1.00
Polysorbate 20	1.00	1.00
Cyclomethicone	4.00	4.00
*Dunaliella salina* extract (in jojoba seed oil)	1.00	0.00
Jojoba seed oil	0.00	1.00
Carbomer	0.80	0.80
Triethanolamine	0.70	0.70
Phenoxyethanol	0.40	0.40
Methyl paraben	0.15	0.15
EDTA	0.10	0.10
TOTAL	100.00	100.00

## Data Availability

The data presented in this study are available on request from the corresponding author.
